# Identification and characterization of bacteria isolated from patients with cystic fibrosis in Jordan

**DOI:** 10.1080/07853890.2022.2131282

**Published:** 2022-10-20

**Authors:** Nid’a Alshraiedeh, Farah Atawneh, Rasha Bani-Salameh, Rawan Alsharedeh, Yara Al Tall, Mohammad Alsaggar

**Affiliations:** a Department of Pharmaceutical Technology, Jordan University of Science and Technology, Irbid, Jordan; b Department of Medical Laboratory Sciences, Jordan University of Science and Technology, Irbid, Jordan; c Department of the Pharmaceutics and Pharmaceutical Technology, Yarmouk University, Irbid, Jordan

**Keywords:** Antimicrobial resistance, antimicrobial resistance genes, biofilm, cystic fibrosis

## Abstract

**Background:**

Notable emergence of multidrug-resistant bacteria has become increasingly problematic worldwide. Most patients with cystic fibrosis (CF) suffer from chronic persistent infections with frequent occurrence of acute exacerbations. Routine screening of bacterial strains, epidemiological characteristics, and resistance patterns are particularly useful for patient management and maintenance of infection control procedures

**Methods:**

In this study, 43 pharyngeal samples were taken from patients with CF. Microbiological bacterial culture and identification, antimicrobial susceptibility testings, biofilm formation, including minimum biofilm eradication concentration (MBEC) and PCR for detecting resistance genes were performed.

**Results:**

All samples were positive for bacterial growth. The predominant species were *Staphylococcus aureus* (41.86%; *n* = 18) and *Pseudomonas aeruginosa* (39.53%; *n* = 17). 30% of isolated bacteria were multidrug-resistant, resisting high concentrations of tested antibiotics. Among the 42 biofilm-forming isolates, 23.8% (*n* = 10) were strong biofilm formers. The occurance of resistance genes varied with *blaKPC* detected in 71% (*n* = 17) of all Gram-negative isolates and *mecA* found in 61% (*n* = 11) of all *S. aureus* strains.

**Conclusions:**

The majority of isolated bacteria were *S. aureus* and *P. aeruginosa*. The high frequency of antimicrobial resistance, the presence of resistance genes, and biofilm formation highlight the challenge in treatment and infection control measures in patients with CF.

**KEY MESSAGES**
- *Staphylococcus aureus* and *Pseudomonas aeruginosa* are the most prevalent pathogens found in patients with CF in Jordan.
- Detection of antimicrobial resistance genes in patients with CF confirms that antimicrobial resistance patterns must always be monitored.
- Biofilm formation significantly increases the tolerance of bacteria to antimicrobial agents.

## Introduction

Cystic fibrosis (CF) is a common autosomal recessive disorder with a frequency of one in 2000–5800 live births in the Middle East and an estimated median survival age of 10–20 years [1]. It affects different body organs, such as the pancreas, the gastrointestinal tract, the salivary glands, and the lungs. However, most deaths are related to respiratory complications. Chronic respiratory infection is a hallmark feature among patients with CF that may manifest early in life and persist for years. Microbial colonization of the lung is predisposed by impaired lung defense against pathogens. Chronic infection has been associated with a progressive decline in lung function and increased mortality rates [2,3]. With the persistence of infection, pathogens adapt continuously, both phenotypically and genotypically, to survive in the abnormal lung environment and tolerate the challenging immune response, as well as antimicrobial treatment [3]. Continuous microbiological screening and monitoring of respiratory pathogens, including their antimicrobial resistancepatterns, is essential for proper therapy and has a significant imapct on life quality improvement of patients with CF. In this study, we describe the prevalence of bacteria isolated from patients with CF in Jordan. We also evaluated their antimicrobial susceptibilities including the underlying resistance genes, their ability to form biofilms, and the effect biofilms pose on antibiotic treatment.

## Materials and methods

### Samples collection

Posterior pharyngeal swabs were collected from clinically stable patients with CF between February 2018 and April 2019. Most of the patients were male (*n* = 26, 60%), with an age range between 5 and 31 years. The swabs were obtained aseptically and immediately inoculated onto blood agar, chocolate agar, MacConkey agar, mannitol salt agar, and cetrimide agar. Plates were incubated aerobically at 37 °C for 18–24 h. All isolates were stored in 1 mL of glycerol stocks at −80° C until further processing.

### Bacterial identification

Bacteria were identified using VITEK 2 Compact Systems (BioMérieux, Marcy-l’Étoile, France). Additionally, several biochemical tests were used to aid in identification such as catalase, coagulase, bacitracin disc, optochin disc, and rapid latex test for streptococcal grouping (Streptex) (R30950501 ZL50, Thermo Fisher Scientific, Waltham, MA, USA) for Gram-positive isolates [4,5], and oxidase, Kligler’s iron agar (KIA), sulphur indol motility (SIM), methyl red/Voges Proskauer (MR-VP), citrate, Remel RapID NF Plus System (REF R8311005, Thermo Fisher Scientific, Waltham, MA, USA), and Remel RapID ONE System (REF R8311006 Thermo Fisher Scientific, Waltham, MA, USA) for Gram-negative isolates [6,7].

### Antimicrobial susceptibility testing

Antibiotic susceptibility testing (AST) was performed using the Kirby-Bauer disc diffusion method and commercial antibiotic discs (Oxoid, Wade Road, Basingstoke, Hants, RG248PW, United Kingdom) according to the CLSI 2018 recommendations [8]. *Staphylococcus aureus* strains were tested for their susceptibilities to ciprofloxacin (5 µg), gentamicin (10 µg), penicillin (10 units), clindamycin (2 µg), erythromycin (15 µg), oxacillin (1 µg), rifampin (5 µg), doxycycline (30 µg) and trimethoprim-sulfamethoxazole (1.25/23.75 μg). *Streptococcus agalactiae* isolate was tested against cefepime (30 µg), gentamicin (10 µg), meropenem (10 µg), levofloxacin (5 µg), clindamycin (2 µg), erythromycin (15 µg), azithromycin (15 µg), ampicillin (10 µg), ofloxacin (5 µg), tetracycline (30 µg), chloramphenicol (30 µg), rifampin (5 µg) and vancomycin (30 µg).

All Gram-negative isolates were tested against ceftazidime (30 µg), ciprofloxacin (5 µg) tobramycin (10 µg), piperacillin-tazobactam (100/10 µg), imipenem (10 µg), gentamicin (10 µg), cefepime (30 µg), aztreonam (30 µg), meropenem (10 µg), amikacin (30 µg) and levofloxacin (5 µg).

### Molecular characterization of antibiotic resistance genes

Gram-negative isolates were tested for carbapenem resistance genes (*blaKPC, blaNDM* and *blaVIM*) [9], and aminoglycoside resistance genes (*armA, rmtB, npmA, rmtE* and* rmtF*) [10] using conventional multiplex PCR, and for extended spectrum ß-lactamase (ESBL) genes (*blaCTX*, *blaTEM* and *blaSHV*) using conventional singleplex PCR [11], according to references.

*S. aureus* isolates were tested for methicillin resistance genes (*mecA* and *mrs*) and erythromycin and clindamycin resistance genes (*ermA, ermB* and* ermC*) [12] using conventional multiplex PCR according to references. *Streptococcus agalactiae* was tested for erythromycin and clindamycin resistance genes (*ermB, ermTR, mefA* and *linB*) using conventional multiplex PCR [13] and erythromycin resistance gene *mefE* using conventional singleplex PCR [14], according to references.

Primers were ordered from the Integrated DNA Technologies (IDT) Company. The GenePro thermal cycler (BIOER Technology, Zhejiang, Hangzhou, Binjiang, China) was used for target amplification, and amplicons were separated via gel electrophoresis at 130 V for 30 min, using a 2% agarose gel and 5 µL PCR product.

### Biofilm formation assay

Biofilm production was quantitatively determined using the tissue culture plate method described previously with slight modifications [15]. Briefly, fresh bacterial suspensions were prepared in trypticase soy broth, supplemented with 1% glucose, and incubated for 24 h at 37° C. A 200 µL inoculum of 0.5 McFarland bacterial suspension was added to each well and incubated for 24 h at 37 °C. After incubation, the good contents were discarded and washed three times with 200 μL phosphate buffer saline to remove planktonic cells. Biofilms were stabilized using 200 μL 30% acetic acid for 15 min and stained with 0.1% crystal violet for 30 min. Two hundred microliters of absolute ethanol were used for dye solubilization, and absorbance was immediately read at optical density OD575 on an ELISA plate reader (BioTek Epoch). Results were interpreted according to reference [15] (Table 1). All isolates were tested in triplicates.

### Minimum biofilm eradication concentration (MBEC)

MBEC for biofilm formers was determined using the Calgary biofilm device (CBD) as previously described with modifications [16]. Briefly, standardized bacterial suspensions were prepared to be equivalent to 10^7^cfu/mL. Each well of the CBD was inoculated with 150 µL and incubated at 37 °C for 48 h with orbital shaking (100 rpm). Upon incubation, the peg lids were washed and challenged using a series of two-fold dilutions of antibiotics, and a starting concentration of 2 mg/mL. Plates were incubated for 24 h at 37 °C. Subsequently, the CBD lids were washed and attached biofilms were released from the peg lids into fresh Mueller-Hinton broth by sonication for 10 min at 37 °C. Cells were allowed to recover for 24 h at 37 °C and MBEC values were determined as the lowest antibiotic concentration that showed no visible growth. All isolates were tested in triplicates.

## Results

### Bacterial identification

**Figure 1.**
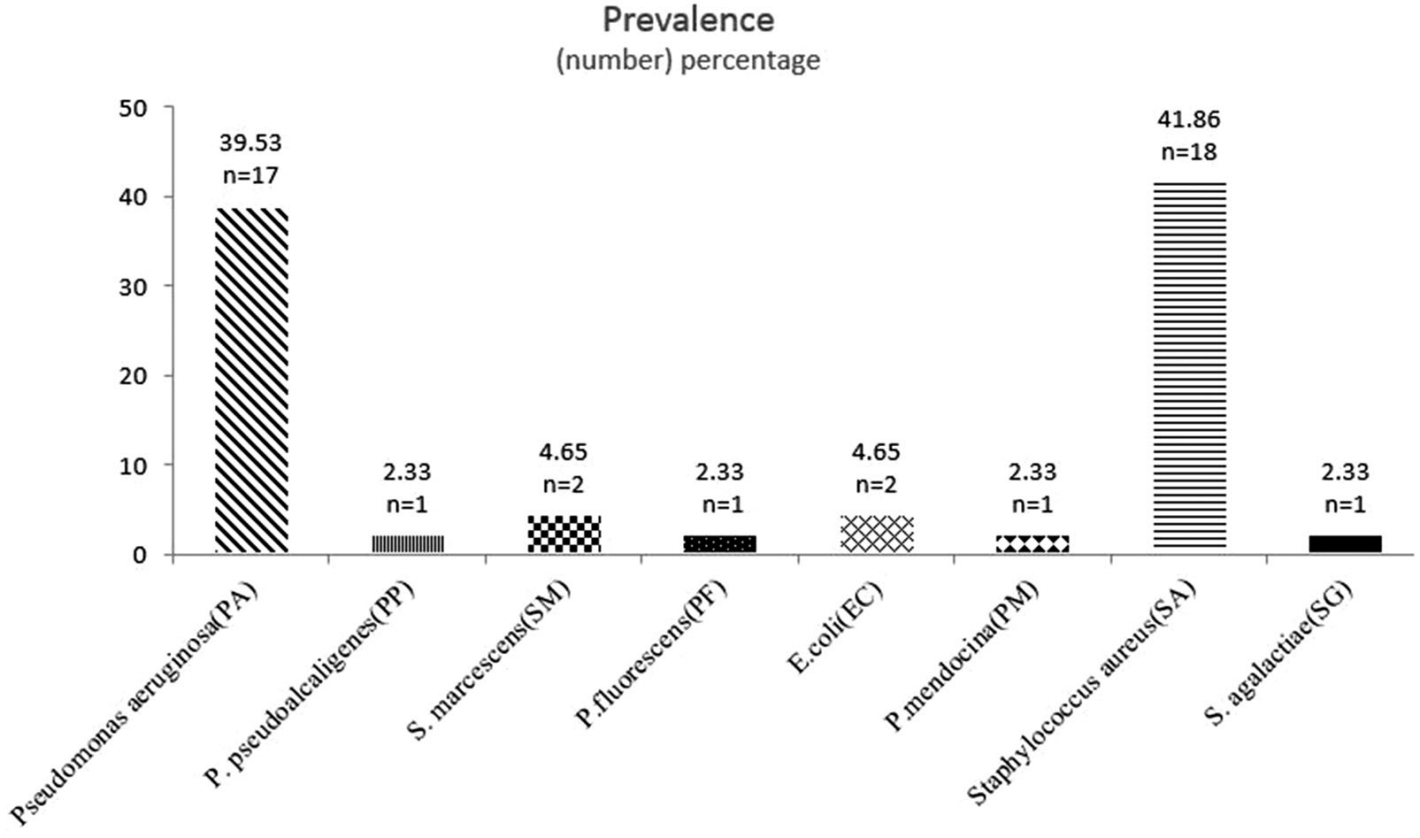
Prevalence of bacterial strains (number and percentage) isolated from patients with cystic fibrosis enrolled in the study.

All samples collected in this study resulted in bacterial growth. Two patients were colonized with both *S. aureus* and *Pseudomonas aeruginosa*. Mixed colonization was also detected in another patient where both *S. aureus* and *Escherichia coli* were detected.24 isolates were Gram-negative (56%) and 19 were Gram-positive (44%). The predominant species was *S. aureus* (41.86%; *n* = 18) followed by *P. aeruginosa* (39.53%; *n* = 17). Less commonly isolated bacteria included *E. coli* (5%; *n* = 2), *Serratia marcescens* (4.65%; *n* = 2), *Pseudomonas pseudoalcaligenes* (2.33%; *n* = 1), *Pseudomonas fluorescens* (2.33%; *n* = 1), *Pseudomonas mendocina* (2.33%; *n* = 1) and *Streptococcus agalactiae* (2.33%; *n* = 1) (Figure 1).

### Antimicrobial susceptibility testing

All Gram-negative bacteria identified were susceptible to piperacillin-tazobactam, ceftazidime, and cefepime. However, low resistance rates of 4% -13% were observed against amikacin, ciprofloxacin, imipenem, aztreonam, meropenem and levofloxacin. All *S. aureus* strains were susceptible to gentamicin, doxycycline, and trimethoprim-sulfamethoxazole. 12 isolates (67%) were not susceptible to oxacillin, 14 (78%) to penicillin, 4 (22%) to clindamycin, 3 (17%) to erythromycin, 2 (11%) to rifampin, and 1 (6%) to ciprofloxacin. *S. agalactiae* was susceptible to meropenem, cefepime and chloramphenicol (Table 2).

**Table 2.** Antibiotic susceptibility percent of isolated bacteria from patients with cystic fibrosis enrolled in the study.

Bacteria antibiotics	Gram-negative bacteria						Gram-positive bacteria	
	*P. aeruginosa*, % (*n*)	*P. pseudoalcaligenes*, % (*n*)	*P. fluorescens*, % (*n*)	*P. mendocina*, % (*n*)	*S. marcescens*, % (*n*)	*E. coli*, % (*n*)	*S. aureus*, % (*n*)	*S. agalactiae*, % (*n*)
CAZ	100 (17)	100 (1)	100 (1)	100 (1)	100 (2)	100 (2)	–	–
TOB	88.2 (15)	100 (1)	100 (1)	100 (1)	100 (2)	50 (1)	–	–
TZP	100 (17)	100 (1)	100 (1)	100 (1)	100 (2)	100 (2)	–	–
IPM	88.2 (15)	100 (1)	100 (1)	100 (1)	100 (2)	100 (2)	–	–
ATM	94.11 (16)	100 (1)	100 (1)	100 (1)	50 (1)	100 (2)	–	–
AK	82.35 (14)	100 (1)	100 (1)	100 (1)	100 (2)	100 (2)	–	–
LEV	100 (17)	100 (1)	100 (1)	100 (1)	50 (1)	50 (1)	–	0
MEM	94.11 (16)	100 (1)	100 (1)	100 (1)	100 (2)	100 (2)	–	100 (1)
FEP	100 (17)	100 (1)	100 (1)	100 (1)	100 (2)	100 (2)	–	100 (1)
CIP	94.11 (16)	100 (1)	100 (1)	100 (1)	50 (1)	50 (1)	94.4 (17)	–
CN	88.2 (15)	100 (1)	100 (1)	100 (1)	100 (2)	100 (1)	100 (18)	0 (0)
(P)	–	–	–	–	–	–	11.11 (4)	–
RA	–	–	–	–	–	–	88.88 (16)	0 (0)
DA	–	–	–	–	–	–	77.77 (14)	0 (0)
E	–	–	–	–	–	–	16.66 (15)	0 (0)
OX	–	–	–	–	–	–	33.33 (6)	–
DO	–	–	–	–	–	–	100 (18)	–
SXT	–	–	–	–	–	–	100 (18)	–
AZM	–	–	–	–	–	–	–	0 (0)
AM	–	–	–	–	–	–	–	0 (0)
OFX	–	–	–	–	–	–	–	0 (0)
TE	–	–	–	–	–	–	–	0 (0)
C	–	–	–	–	–	–	–	100 (1)
VA	–	–	–	–	–	–	–	0 (0)

Abbreviations. CAZ: ceftazidime; TOB: tobramycin; TZP: piperacillin-tazobactam; IPM; imipenem; ATM: aztreonam; AK: amikacin; LEV: levofloxacin; MEM: meropenem; FEP: cefepime; CIP: ciprofloxacin; CN: gentamicin; P: penicillin; RA: rifampin; DA: clindamycin; E: erythromycin; OX: oxacillin; DO: doxycycline; SXT: trimethoprim-sulfamethoxazole; AZM: azithromycin; AM: ampicillin; OFX: ofloxacin; TE: tetracycline; C: chloramphenicol; VA: vancomycin; –: not tested.

Multidrug resistance, defined as non-susceptibility to at least one antimicrobial agent of at least three classes, was observed in 13 isolates (30%).

### Quantitative biofilm formation assay

Almost all isolated bacteria produced some amount of biomass. About 23.26% of isolates (10 isolates) were strong, 23.26% (10 isolates) were moderate, and 51.16% (22 isolates) were weak biofilm formers (Table 3).

**Table 3.** Biofilm formation profile of bacteria isolated from patients with cystic fibrosis enrolled in the study.

Bacterial isolates	Biofilm formation patterns			
	Strong biofilm formers, *n* (%)	Moderate biofilm formers, *n* (%)	Weak biofilm formers, *n* (%)	Non biofilm producer, *n* (%)
*Staphylococcus aureus*	0 (0)	6 (33.33)	12 (66.66)	0 (0)
*Pseudomonas aeruginosa*	8 (47)	4 (23.5)	5 (29.4)	0 (0)
*P. pseudoalcaligenes*	1 (100)	0 (0)	0 (0)	0 (0)
*S. marcescens*	0 (0)	0 (0)	2 (100)	0 (0)
*E. coli*	0 (0)	0 (0)	1 (50)	1 (50)
*S. agalactiae*	0 (0)	0 (0)	1 (100)	0 (0)
*P. fluorescens*	0 (0)	0 (0)	1 (100)	0 (0)
*P. mendocina*	1 (100)	0 (0)	0 (0)	0 (0)
Total	10 (23.26)	10 (23.26)	22 (51.16)	1 (2.33)

### Minimum biofilm eradication concentration (MBEC)

Drastic increases in MBEC values were noticed in most biofilm-forming isolates. While all *P. aeruginosa* strains tested susceptible to ceftazidime and levofloxacin when grown as planktonic, 94% (*n* = 16) and 35% (*n* = 6) required MBEC values ≥500 µg/mL, respectively. All strains required similar high MBEC values when tested against aztreonam, while 94% (*n* = 16) reacted susceptible to it in the planktonic form. Meropenem and amikacin susceptibility was observed in 94% (*n* = 16) and 82% (*n* = 14) of these isolates, with MBEC values of ≥500 µg/mL in 94%(*n* = 16) and 82%(*n* = 14), respectively. *Pseudomonas* strains other than *aeruginosa*, reacting susceptible to meropenem, aztreonam, and ceftazidime in the planktonic form, all required MBEC values ≥500 µg/mL. 88% of our *S. aureus* isolates were readily eradicated with gentamycin and MBEC values ranging from 0.9765–12.5 µg/mL. 16 isolates of *S. aureus* required MBEC values ≥500 µg/mL for doxycycline, while previously all tested susceptible to it in the planktonic form. Similar observations were observed with less frequently isolated organisms (Table 4). The biofilm produced by *S. agalactiae* was eradicated using cefepime, meropenem and levofloxacin with MBEC values of 0. 9765 µg/µL and 3.906 µg/µL, respectively.

**Table 4.** MBEC (µg/mL) of tested antibiotics for bacteria isolated from patients with cystic fibrosis enrolled in the study.

Isolate #	Tested antibiotics							
	AK	MEM	ATM	CAZ	LEV	CN	DO	FEP
PA1	≥500	≥500	≥500	≥500	0.015625	–	–	–
PA2	≥500	≥500	≥500	≥500	≥500	–	–	–
PA3	≥500	≥500	≥500	≥500	0.00195	–	–	–
PA4	≥500	≥500	≥500	≥500	0.015625	–	–	–
PA5	≥500	≥500	≥500	≥500	≥500	–	–	–
PA6	≥500	≥500	≥500	≥500	≥500	–	–	–
PA7	≥500	≥500	≥500	≥500	≥500	–	–	–
PA8	≥500	≥500	≥500	≥500	0.03125	–	–	–
PA9	≥500	0.3125	≥500	≥500	0.0009765	–	–	–
PA10	≥500	≥500	≥500	≥500	0.00195	–	–	–
PA11	≥500	≥500	≥500	≥500	0.0625	–	–	–
PA12	0.3125	≥500	≥500	≥500	≥500	–	–	–
PA13	0.039	≥500	≥500	0.0009765	0.0009765	–	–	–
PA14	≥500	≥500	≥500	≥500	0.0078	–	–	–
PA15	0.0625	≥500	≥500	≥500	0.0625	–	–	–
PA16	≥500	≥500	≥500	≥500	≥500	–	–	–
PA17	≥500	≥500	≥500	≥500	0.0156	–	–	–
PP1	0.00781	≥500	≥500	≥500	≥500	–	–	–
PM1	0.03125	≥500	≥500	≥500	≥500	–	–	–
PF1	≥500	≥500	≥500	≥500	0.0009765	–	–	–
EC1	≥500	≥500	≥500	≥500	≥500	–	–	–
EC2	≥500	≥500	≥500	≥500	0.00195	–	–	–
SM1	≥500	≥500	≥500	0.0009765	0.00195	–	–	–
SM2	≥500	≥500	≥500	≥500	≥500	–	–	–
SA1	–	–	–	–	–	0.0009765	≥500	–
SA2	–	–	–	–	–	0.0009765	≥500	–
SA3	–	–	–	–	–	0.03125	≥500	–
SA4	–	–	–	–	–	0.0009765	≥500	–
SA5	–	–	–	–	–	0.03125	0.003906	–
SA6	–	–	–	–	–	≥500	≥500	–
SA7	–	–	–	–	–	0.0625	≥500	–
SA8	–	–	–	–	–	0.003906	≥500	–
SA9	–	–	–	–	–	0.015625	0.0009765	–
SA10	–	–	–	–	–	0.125	≥500	–
SA11	–	–	–	–	–	0.03125	≥500	–
SA12	–	–	–	–	–	0.00195	≥500	–
SA13	–	–	–	–	–	0.015625	≥500	–
SA14	–	–	–	–	–	≥500	≥500	–
SA15						0.00195	≥500	
SA16						0.0625	≥500	
SA17						0.000975	≥500	
SA18						0.000975	≥500	
SG		0.00391			0.015625			0.0009765

Abbreviations. PA: *P. aeruginosa*; PP: *P. pseudoalcaligenes*; PM: *P. mendocina*; PF: *P. fluorescens*; EC: *E. coli*; SM: *S. marcescens*; SA: *S. aureus*; SG: *S. agalactiae*; AK: amikacin; MEM: meropenem; ATM: aztreonam; CAZ: ceftazidime; LEV: levofloxacin; CN: gentamicin; DO: doxycycline; FEP: Cefepime; -: not tested.

### Antimicrobial resistance genes

In all Gram-negative isolates tested, carbapenemase genes were detected more frequently than ESBL genes. The predominant carbapenemase was blaKPC found in 71% (*n* = 17), followed by blaNDM and blaVIM, found in 46% (*n* = 11) and 29% (*n* = 7) respectively. A total of 5 isolates carried blaCTX and blaSHV, while none of them carried blaTEM or any of the tested aminoglycoside resistance genes.

Among all S. aureus strains, mecA was detected in 61% (*n* = 11). mrsA/B, ermA, ermB and ermC were detected at lower frequencies of 11% (*n* = 2), 22% (*n* = 4), 11% (*n* = 2), and 44% (*n* = 8). The *S. agalactiae* isolate was not found to contain any of the resistance genes (Table 5). Representative gel electrophoresis images of the detected genes are shown in Supplementary Figures 1–4.

**Table 5.** Frequency (percentage and number) of antibiotic resistance genes among Gram-negative and Gram-positive bacteria isolated from patients with cystic fibrosis enrolled in the study.

Bacterial isolates	Resistance gene in Gram-negative bacteria						Resistance gene in Gram-positive bacteria									
	Frequency of carbapenem resistance genes, *n* (%)			Frequency of ESBL resistance genes, *n* (%)			Frequency of methicillin resistance genes, *n* (%)			Frequency of erythromycin and clindamycin resistance genes, *n* (%)						
	*BlaKPC*	*blaNDM*	*BlaVIM*	*BlaTEM*	*Bla CTX*	*Bla SHV*	*nuc*	*mecA*	*MRS (A/B)*	*ermA*	*ermB*	*ermC*	*ermTR*	*mefA*	*mefE*	*linB*
*P. aeruginosa*	11 (65)	7 (41)	4 (24)	0 (0)	3 (18)	5 (29)	–	–	–	–	–	–	–	–	–	–
*P. pseudoalcaligenes*	1 (100)	1 (100)	1 (100)	0 (0)	0 (0)	0 (0)	–	–	–	–	–	–	–	–	–	–
*S. marcescens*	2 (100)	1 (50)	2 (100)	0 (0)	0 (0)	0 (0)	–	–	–	–	–	–	–	–	–	–
*E. coli*	2 (100)	2 (100)	0 (0)	0 (0)	2 (100)	0 (0)	–	–	–	–	–	–	–	–	–	–
*P. fluorescens*	0 (0)	0 (0)	0 (0)	0 (0)	0 (0)	0 (0)	–	–	–	–	–	–	–	–	–	–
*P. mendocina*	100 (1)	0 (0)	0 (0)	0 (0)	0 (0)	0 (0)	–	–	–	–	–	–	–	–	–	–
*S. aureus*	–	–	–	–	–	–	17 (94)	11 (61)	2 (11)	4 (22)	2 (11)	8 (44)	–	–	–	–
*S. agalactiea*	–	–	–	–	–	–	–	–	–	–	0 (0)		0 (0)	0 (0)	0 (0)	0 (0)
Number of isolates (%)	17 (71)	11 (46)	7 (29)	0 (0)	5 (21)	5 (21)	17 (89.5)	11 (63.2)	2 (15.8)	4 (21.1)	2 (15.8)	8 (42.1)	0 (0)	0 (0)	0 (0)	0 (0)

## Discussion

CF is an autosomal recessive disorder resulting from a mutation in the cystic fibrosis transmembrane conductance regulator gene (CFTR), which plays a significant role in controlling fluid and electrolyte transmission across epithelial membranes. Consequently, the viscosity of mucus membranes in multiple organs such as the lungs, the pancreas, the liver, the gut, and others, provides a suitable environment for the colonization of different microorganisms such as *S. aureus*, *P. aeruginosa*, *Burkholderia* spp. *Stenotrophomonas maltophilia*, and others [3].

The prevalence and characterization of respiratory pathogens isolated from patients with CF have been frequently studied and require routine monitoring. *Staphylococcus aureus* represented 42% (*n* = 18) of the in here isolated bacteria, followed by *P. aeruginosa* with 40% (*n* = 17). Findings that are in agreement with previous reports from France, which list *S. aureus* as the most frequently isolated organism in paediatrics and adults in 35% and 38%, respectively, followed by *P. aeruginosa* which was isolated in 31% of investigated adults [17]. A similar prevalence was reported in Brazil, where *S. aureus* was isolated from 50% of a tested cohort of patients with CF, and *P. aeruginosa* in 35% [18]. In a different work from Brazil, *P. aeruginosa* and *S. aureus* were reported in 36.2% and 28.9% of the individuals tested, respectively [18]. In Tehran, frequency variations were reported, where *P. aeruginosa* accounted for 55.5% of all positive cultures, followed by *S. aureus* (15.6%) and Klebsiella *pneumoniae* (11.7%) [19]. This difference in the predominance of microorganisms may be attributed to various factors, such as the age of the population. In most cases of cystic fibrosis, *S. aureus* is the first pathogen that infects the lungs and the most common pathogen isolated from patients with CF. During the course of the illness, more virulent and challenging bacteria colonisze the lung, such as *P. aeruginosa,* *methicillin‐resistant S. aureus*, *S. maltophilia*, *non-tuberculous mycobacteria*, and others [3].

Increasing resistance rates against frequently used antibiotics is a global concern. As bacterial infection advances in patients with CF, it becomes more difficult to achieve the same degree of clinical response with antibiotic therapy, and lung infections become more refractory to treatment. Recurrent infections require more frequent antibiotic treatments, increasing the chance of emerging antimicrobial resistance. Hence, routine AST is of utmost importance to monitor antibiotic resistance profiles. Multidrug resistance was found in 30% (*n* = 13) of the isolates tested here. All *P. aeruginosa* isolates were susceptible to ceftazidime, piperacillin-tazobactam, cefepime, and levofloxacin. Attention was paid to aztreonam and tobramycin, two antibiotic agents recommended for the treatment of *P. aeruginosa* infections [20]. Low resistance rates of 6% (*n* = 1) and 12% (*n* = 2) were observed for these two agents, respectively. Similarly, low rates were also observed for meropenem (6%; *n* = 1), gentamycin (12%; *n* = 2) and imipenem (12%; *n* = 2). In comparison to other regions, these low rates raise reason for hope, as higher resistance rates are commonly seen in patients with CF. Reports from Brazil reveal >50% resistance to gentamycin and imipenem among mucoid and nonmucoid strains of *P. aeruginosa.* However, more than 75% of both phenotypes were susceptible to ceftazidime, ciprofloxacin, and piperacillin, and even 100% were susceptible to meropenem [18]. Northern Europe reported that more than 50% of *P. aeruginosa* isolates were resistant to penicillin (ticarcillin, piperacillin and piperacillin-tazobactam), 59% to ceftazidime, 46% to amikacin, 27% ciprofloxacin, 20% to carbapenems and 16% to tobramycin [21].

Among our isolated *S. aureus* strains, the highest resistance rates were observed in penicillin (89%; *n* = 16), followed by erythromycin (83%; *n* = 15) and oxacillin (67%; *n* = 12). Methicillin-resistant *S. aureus* (MRSA) identification and diagnosis in the clinical microbiology environment are critical for both determining effective treatment for individual patients and MRSA surveillance. MRSA strains are defined by the detection of the *mecA* gene which encodes PBP 2a; an altered penicillin-binding protein that has a low affinity for β-lactam antibiotics. The prevalence of MRSA, defined by detection of the *mecA* via PCR, was detected among 61% (*n* = 11) of *S. aureus* isolates. The slight difference between isolates resistant to oxacillin and *mecA*-positive isolates could be due to the presence of *mecA* homologous, such as *mecC*, emphasizing the need to search for both genetic resistance genes when identifying MRSA. Recently, isolates positive for MRSA but negative for mecA were discovered and found to harbour mecC, a homolog of mecA [22]. All *S. aureus* isolates were susceptible to gentamicin, doxycycline, and trimethoprim-sulfamethoxazole. In contrast to our findings, low rates of MRSA were reported in different studies conducted in different countries [23–25].

When investigating underlying resistance elements, carbapenemases were detected more frequently compared to the ESBL genes. All carbapenemase genes were detected in our Gram-negative isolates. *blaKPC* and *blaNDM* had the highest rates detected in 71% (*n* = 17) and 46% (*n* = 11), respectively. *blaVIM* was detected in 29% (*n* = 7) of Gram-negative organisms. However, not all ESBL genes tested here were detected. *blaSHV* and *blaCTX* were seen at equal frequencies of 21% (*n* = 5), but none of the isolates harboured *blaTEM*. All numbers of worrying concerns compared to other regions. For instance, in a study conducted in Tehran, CTX-M and VIM were reported to be as low as 19% and 3%, respectively [26]. Another work from Iran reported that *ESBL* gene or KPC were not detected in *P. aeruginosa* strains [27]. In our study, high rates of MRSA were observed among *S. aureus* isolates, as 61% harboured *mecA*. Macrolide, lincosamide, and streptogramin-B, collectively called MLS-B, were detected at lower frequencies among *S. aureus* isolates. A difference in frequencies of resistance genes compared to other regions is reported. A study carried out in the Czech Republic reported that 39% of isolated strains of *S. aureus* harbour *mecA*. MLS-B genes were also detected in lower percentages since *ermC* and *ermA* were found in 17.8% and 16% of these isolates, respectively [24]. Variations can be attributed to differences in sample size.

In addition to the crucial role of the acquisition of resistance genes in the development of antimicrobial resistance, the virulent phenotypic trait of biofilm formation significantly contributes to the persistence of infection and antibiotic resistance in patients with CF. Bacteria protected by biomass withstand stressful conditions and gain protection against antimicrobials and host defenses. Almost all isolated organisms produced some amount of biomass with varying degrees. Our results come in agreement with a Spanish study that showed that 94.1% of MRSA isolates were biofilm formers [28]. Biofilm production was also reported at 76.5%, 67% and 72.5% of *P. aeruginosa*, *S. aureus* and *K. pneumoniae* isolated from patients with CF in Brazil, respectively [29].

When evaluating the effect of biofilm formation on antimicrobial affectivity, more than 80% of Gram-negative bacteria exhibited a considerable high MBEC (≥500 µg/mL) to ceftazidime, aztreonam, meropenem, and amikacin, while 70% of these exhibited a susceptible pattern to the same antibiotics in the disc diffusion method. Generally, our isolates grown in biofilms tolerated higher concentrations of antibiotics, which required high MBEC values to eradicate these compared to the antibiotic concentrations required to inhibit bacterial growth in the planktonic form.

## Conclusion

Our findings showed a high prevalence of *S. aureus* and *P.* aeruginosa among patients with CF in Jordan. Biofilm formation significantly increased the tolerance of bacteria to most of the antibiotics tested. In addition,resistance genes were also detected. Periodic monitoring of clinically relevant pathogens in CF along with virulent characteristics, including biofilm formation, can impact the treatment strategies and their outcomes. The spread of MRSA, as well as ESBL and carbapenemase-producing bacteria, dramatically affects the prognosis of treatment in CF. Continuous surveillance work is required to define and implement up-to-date treatment options and control measures.

## Ethical approval

The study was approved by the institutional review board of the Jordan University of Science and Technology, Irbid, Jordan. Informed signed consent was obtained.

## Author contributions

Nid’a Alshraiedeh has substantially contributed to conceptualization; design of the study; performing and supervising laboratory works, Data curation, Data analysis, interpretation of the data, funding acquisition, project administration; resources, and writing the original draft.

Farah Atawneh has substantially contributed to performing laboratory work, data acquisition, data analysis, interpretation of data, and drafting the manuscript.

Rasha Bani-Salameh has substantially contributed to the interpretation of data, drafting the manuscript, and critically revising it for intellectual content.

Rawan Alsharedeh, Yara Al Tall, and Mohammad Alsaggar have substantially contributed to the analysis, interpretation of data, and revising of the manuscript.

## Supplementary Material

Supplemental Material

Supplemental Material

## Data Availability

All data generated or analyzed during this study are included in this article. Interpretation of optical density data for detection of biofilm formation [15]. OD: optical density at 575 nm. Optical density cut-off value (ODc) = average OD of negative control + 3 × standard deviation (SD) of the negative controls.
