# Supplementary material for: Identification and characterization of bacteria isolated from patients with cystic fibrosis in Jordan
Source: Ann Med. 2022 Oct 20;54(1):2796–804. doi: 10.1080/07853890.2022.2131282 (PMC9586617; doi:10.1080/07853890.2022.2131282)
Supplement: Supplemental Material [file IANN_A_2131282_SM9680.docx]

**SUPPLEMENTARY**


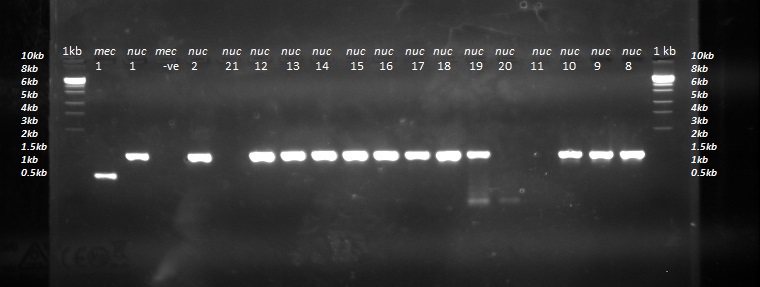


**A**

**B**


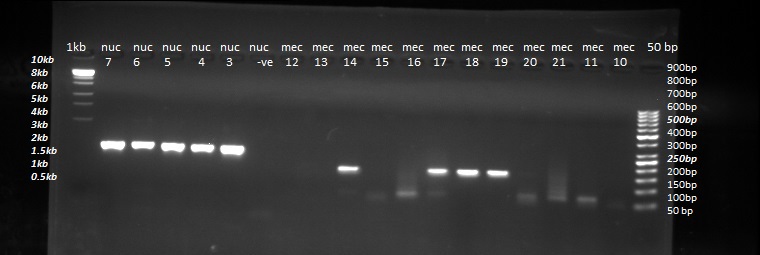


**C**


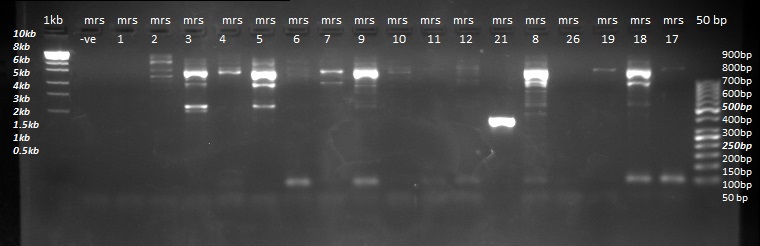


**Figure 1:** Representative gel electrophoresis images for detection of *nuc, mec* and *mrs*. Each lane is labled with name af gene and isoalte ID.


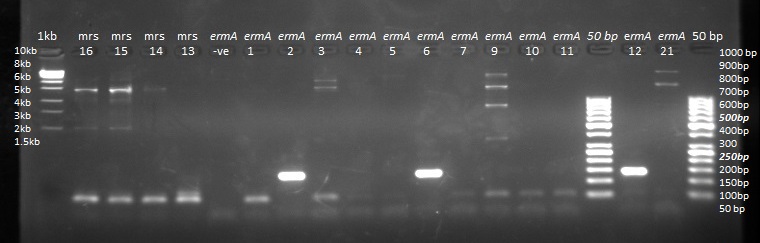


**B**

**A**


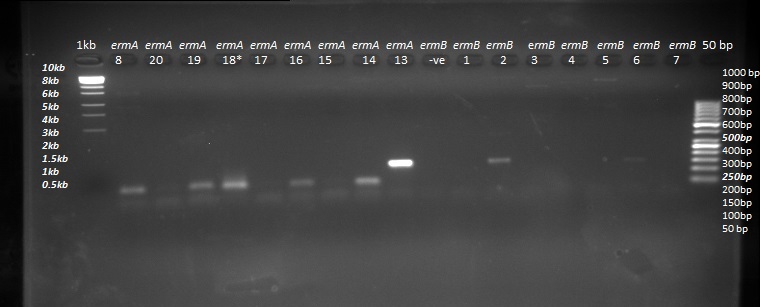


**C**


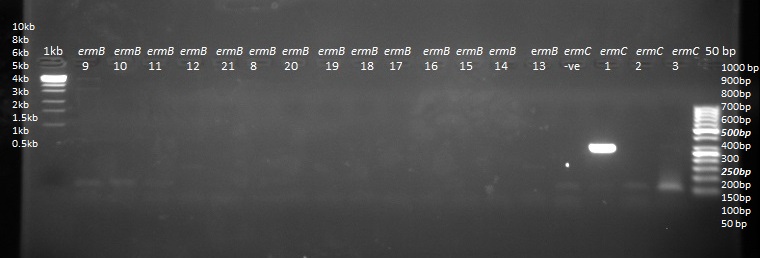


**D**


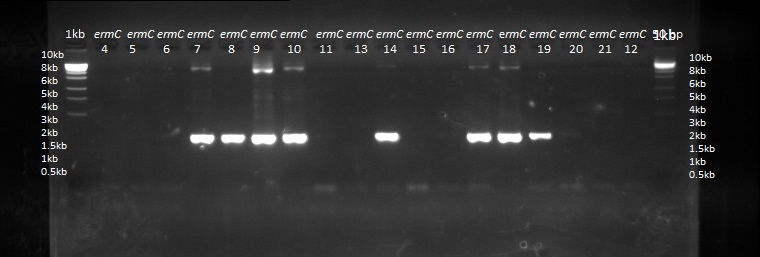


**Figure 2:** Representative gel electrophoresis images for detection of *ermA, ermB*and *ermC*.. Each lane is labled with name af gene and isoalte ID.


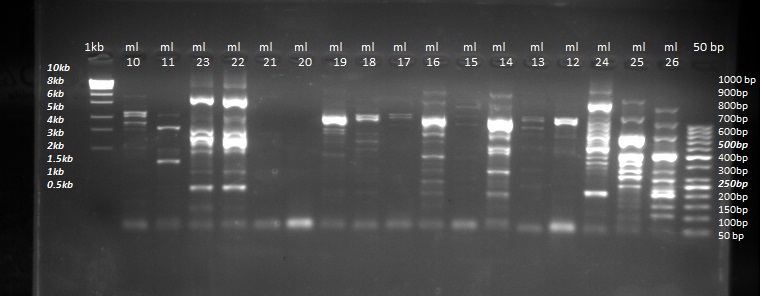


**Figure 3:** Representative gel electrophoresis images for detection of *blaKPC, blaNDM , and blaVIM*. Name af gene and isoalte ID are shown in Each lane.


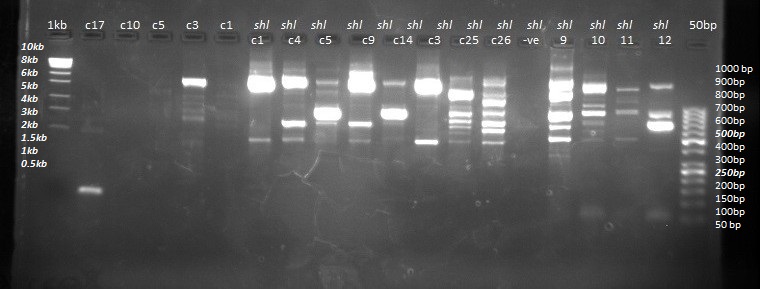


**B**

**A**


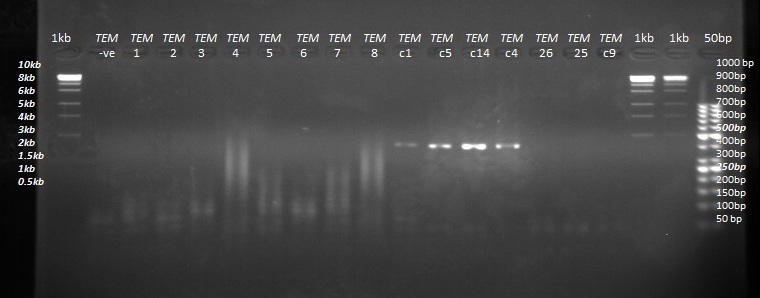


**C**


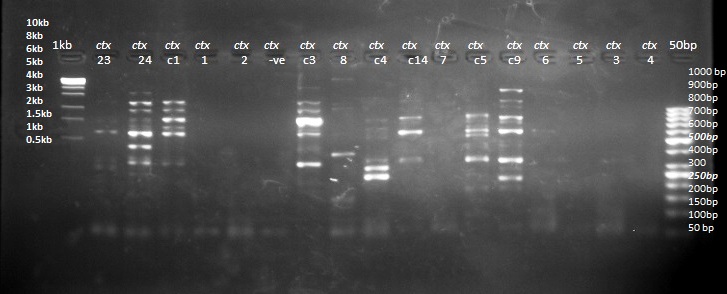


**Figure 4:** Representative gel electrophoresis images for detection of *blaSHV, blaTEM* and *blaCTX*. Each lane is labled with name af gene and isoalte ID.
